# The moderating effect of work-related musculoskeletal disorders in relation to occupational stress and health-related quality of life of construction workers: a cross-sectional research

**DOI:** 10.1186/s12891-024-07216-4

**Published:** 2024-02-16

**Authors:** Soo Jeong, Byoung-Hee Lee

**Affiliations:** 1 Department of Physical Therapy, Chung-Ang University Hospital, Seoul, Republic of Korea; 2 Department of Physical Therapy, Sahmyook University, Hwarang-Ro, Nowon-Gu, 815 Seoul, Republic of Korea

**Keywords:** Work-related musculoskeletal disorder, Health-related quality of life, Occupational stress, Construction workers

## Abstract

**Background:**

This study aimed to investigate work-related musculoskeletal disorders (WMSDs), occupational stress, and health-related quality of life (HRQoL); identify the factors that affect HRQoL; and investigate the moderating effects of WMSDs on occupational stress and HRQoL.

**Methods:**

The participants were construction workers who had worked in the construction industry for over three months. A total of 178 construction workers voluntarily participated and anonymously completed the musculoskeletal symptoms questionnaire, the Korean Occupational Stress Scale, short-form 36. The moderation effect of WMSDs on occupational stress and HRQoL were analyzed by Haye’s Process Macro Model.

**Results:**

The results of the study showed that 96 subjects (53.9%) had WMSDs, and the most common pain site was the lower back (33.3%). The group with WMSDs had higher occupational stress than did the group without WMSDs (*p* < 0.01). Compared with the group without WMSDs, the group with WMSDs displayed significant differences in HRQoL (*p* < 0.001). Furthermore, the factor affecting HRQoL was WMSDs (*p* < 0.001). In the impact of occupational stress on HRQoL, WMSDs had a significant moderating effect (*p* < 0.001).

**Conclusion:**

The results of this study indicate that construction workers’ WMSDs significantly impact occupational stress and HRQoL, and WMSDs have a significant moderating effect on the relationship between occupational stress and HRQoL. Therefore, to improve the HRQoL of workers in the construction industry, it is necessary to develop methods to reduce occupational stress and prevent and treat WMSDs.

## Background

Work-related musculoskeletal disorders (WMSDs) are conditions that affect the muscles, tendons, joints, nerves, and supporting blood vessels that occur during work-related activities, such as working in the same position for long periods of time, overexertion in carrying and lifting heavy objects, repetitive tasks, awkward body postures, and whole-body vibrations [1]. Furthermore, WMSDs include muscle and tendon strain, ligament sprains or tears, carpal tunnel syndrome, herniated disc, and connective tissue damage [2]. The proportion of all occupational diseases in the Republic of Korea has increased significantly from 6,715 in 2018 to 9,438 in 2019 [3]. Notably, WMSDs represent a significant health problem worldwide with important socio-economic consequences [4]. Indeed, they affect approximately one-third of the global population, representing one of the most important causes of chronic disability, sick leave absence, reduced work productivity, and lower quality of life [5].

The construction industry has the dubious distinction of being the most injury-prone and one of the most hazardous industries in the world; moreover, it has the second-highest manufacturing industry, accounting for 12.4% of work-related musculoskeletal diseases after the manufacturing industry in the Republic of Korea [6]. This includes constantly using machinery and power tools, working on elevated scaffolds, and manually handling heavy construction materials [7]. Additionally, ever-changing work environments, strict timeframes, and the employment of unskilled workers for daily wages expose workers to unforeseen and unfamiliar hazards at construction sites. They face various types of job-related safety hazards, ergonomic hazards from lifting heavy loads, hazards related to poor or insecure employment conditions, and job-related stress, placing their health and often living at risk. Notably, it is not just workers’ safety at risk but also their health and well-being [8].

Most of the occupational diseases diagnosed in construction workers are multifactorial in nature [9]. Specifically, stressful conditions in the workplace may lead to detrimental physical, psychological, and social changes, which in turn may lead to health-related problems such as altered body composition [10], depression, anxiety, sleep disorders and stress [11]. Moreover construction workers experience fatigue and stress because their work schedules change flexibly depending on the process conditions, and regular holidays and vacations are difficult to guarantee at construction sites in the Republic of Korea [12].

Occupational stress is an adverse physical and emotional reaction that occurs when job requirements do not match employees' abilities, resources, and needs [13]. Notably, work-related psychosocial factors such as workload and closing pressure can affect WMSDs [10], which affects the quality of life [8, 14]. Research was carried out in the foreign countries to explore the relationship between occupational stress and WMSDs among construction workers [8, 15]. Additionally, factors affecting the quality of life of construction workers were investigated [8, 16]. However, research on occupational stress and health-related quality of life (HRQoL) among construction workers in Korea remains scarce. Therefore, the present study confirmed the effects of WMSDs on occupational stress and HRQoL in construction workers and attempted to determine the moderating effect of WMSDs on the relationship between occupational stress and HRQoL.

## Methods

### Participants and recruitment method

The sample size was calculated using G*Power Version 3.1.9.7 (Franz Faul, University Kiel, Germany, 2020). Based on regression analysis, the primary method for testing the program's effectiveness, the required sample size to maintain an effect size of 0.15, α err prob of 0.05, power of 0.95, and number of predictors as 8 is 160. In this study, 200 construction workers were recruited in considering a 20% dropout rate, recovery rate, and an insufficient number of responses. The inclusion criteria were those who had been in the construction industry for more than three months, understood the purpose of this study, and agreed to participate. The exclusion criteria were as follows: those who did not agree to participate in the study or those who did not respond sufficiently. The purpose and necessary matters of the study were fully explained to all participants, and the experiment was conducted after they signed a consent form.

### Experimental procedures

This cross-sectional study was conducted using continuous sampling at three construction sites in Seoul and Gyeonggi-do, Korea, from January 2021 to March 2021. This study was conducted with consent after visiting a construction company in Gyeonggi-do and explaining the purpose of the study to the general manager. The general manager received a recruitment notice prepared by the researcher and posted it at the construction site. The general manager confirmed that participants who expressed their intention to participate after seeing the recruitment notice met the inclusion criteria. The confirmed participants finally heard about the purpose of the study from the general manager and willingly agreed to participate, filling out the questionnaire voluntarily. The completed questionnaire was a self-administered questionnaires about WMSDs, occupational stress, and HRQoL. Questionnaires were distributed and collected either by mail to the general manager or by visiting the construction site. A total of 200 questionnaires were distributed, and 180 were collected. Of these, 178 were analyzed, except for two that disagreed with the consent column, had insufficient responses, or were inappropriate.

### Outcome Measurements

#### WMSDs

The status of WMSDs was investigated using the guidelines for investigating harmful factors of musculoskeletal burden provided by the KOREA OCCUPATIONAL SAFETY and HEALTH AGENCY. The prevalence survey of WMSDs among construction workers aimed to identify if they had experienced uncomfortable symptoms such as pain or numbness in specific body parts—namely the neck, shoulder, arm/elbow, hand/wrist/finger, low back, or leg/foot—over the past year. The participants selected only the most painful part out of the 6 parts. The severity of symptoms was not assessed.

### Occupational Stress

In this study, the Korean Occupational Stress Scale developed by the KOREA OCCUPATIONAL SAFETY & HEALTH AGENCY was used to measure occupational stress [17]. The survey consisted of 43 questions and eight areas. The sub-areas of the tool were physical environment (three questions), job demand (eight questions), insensitive job control (five questions), interpersonal conflict (six questions), job security (four questions), organizational system (seven questions), lack of awards (six questions), and occasional climate (four questions). As an occupational stress evaluation method, scores in the lower eight areas and 100 points were converted, and the average value was obtained and used.

### HRQoL

The Short Form Health Survey (SF-36) was used to evaluate the HRQoL. The SF-36 is a questionnaire developed to measure health status and consists of the Physical Component Summary and Mental Component Summary; the average value of these two factors is called Global Health (Ware et al., 2007). Physical Functioning, Physical Role, Bodily Pain, and General Health are subscales of the Physical Component Summary, while Vitality, Social Functioning, Emotional Role, and Mental Health are subscales of the Mental Component Summary. The scores for all eight subscales were weighted according to the responses and summed. Each item's score was then converted to a scale of 0–100 points; a higher score indicated a healthier subject. A Cronbach's α of 0.82 for the SF-36 was reported [18].

### Statistical analysis

For all tasks and statistics used in the analysis method of this study, means and standard deviations were calculated using SPSS (version 22.0; IBM, Armonk, NY, U.S.A). The general characteristics of the participants and WMSDs were analyzed using frequency analysis, and an independent sample t-test was conducted to analyze the differences in occupational stress and HRQoL according to WMSDs. Dummy variable regression analysis was conducted to identify factors affecting HRQoL. Data were analyzed using the SPSS PROCESS macro (Model 1), developed by Hayes (2013), to verify the moderating effect of WMSDs on occupational stress in relation to HRQoL. All statistical significance levels were set at 0.05.

## Results

### Demographics and occupational characteristics of construction workers

Table 1 shows the demographics and prevalence of WMSDs among construction workers. This study included 156 men (87.6%) and 22 women (12.4%). The age group with the highest proportion consisted of 53 individuals (29.8%) aged 45–54 years. Additionally, 120 (67.4%) participants were technical workers. Regarding the working period, 39 individuals (21.9%) had worked for more than 20 years. Furthermore, 83 (46.6%) worked five days a week, and 91 (51.1%) worked within an eight-hour workday.

**Table 1:** Demographics and occupational characteristics of construction workers

variables	categories	*N* (%)
Sex	Male	156 (87.6)
	Female	22 (12.4)
Age	under 35	50 (28.1)
	35–44	35 (19.7)
	45–54	53 (29.8)
	55 and over	40 (22.5)
Marital status	Single	126 (70.8)
	Married	50 (28.1)
	Others	2 (1.1)
Occupation	blue-collar	120 (67.4)
	white-collar	58 (32.6)
Job experience (years)	< 5	37 (20.8)
	5 ≤ , < 10	36 (20.2)
	10 ≤ , < 15	35 (19.7)
	15 ≤ , < 20	31 (17.4)
	20 ≤	39 (21.9)
Working day per week (day)	5	83 (46.6)
	6	42 (23.6)
	Process situation	53 (29.8)
Working hours per day	≤ 8	91 (51.1)
	≤ 10	52 (29.2)
	10 <	35 (19.7)

### Prevalence of work-related musculoskeletal disorder and pain site

According to the questionnaire, 96 (53.9%) participants experienced pain, numbness, and other uncomfortable work-related symptoms in the past 12 months, including neck, shoulder, arm/elbow, hand/wrist/finger, low back, and leg/foot. In particular, the back was the most affected area among the participants, with 32 patients (33.3%) suffering from back pain. Table 2 shows the prevalence of WMSDs symptoms in various body parts.

**Table 2:** Prevalence of work-related musculoskeletal disorder and pain site

variables	categories	*N* (%)
WMSDs	Yes	96 (53.9)
	No	82 (46.1)
Pain site (96)	Neck	22 (22.9)
	Shoulder	17 (17.7)
	Elbow / forearm	2 (2.1)
	Wrist/Finger	6 (6.3)
	Lower back	32 (33.3)
	Leg / Ankle	17 (17.7)
Duration of pain (96)	< 1 day	11 (11.5)
	1 day ≤ , < 1 week	70 (72.9)
	1 week ≤ , < 1 month	9 (9.4)
	1 month ≤ , < 6 month	6 (6.3)
Treatment (96)	Yes	77 (80.2)
	No	19 (19.8)

### Effects of work-related musculoskeletal disorders on occupational stress

Table 3 shows the effects of work-related musculoskeletal disorders on occupational stress. Notably, there was a significant difference in occupational stress among construction workers according to the presence or absence of WMSDs (*p* < 0.01). Moreover, workers in the construction industry with WMSDs experienced higher stress regarding job demands (*p* < 0.001) and job insecurity (*p* < 0.01) than did those without WMSDs.

**Table 3:** Effects of work-related musculoskeletal disorders on occupational stress

Scales	Without WMSDs symptoms	With WMSDs symptoms	**t*(p)***
	Mean ± SD	Mean ± SD	
Physical environment	47.3 ± 20.1	48.8 ± 19.1	0.526(0.599)
Job demand	43.0 ± 12.7	51.4 ± 12.5	**4.449(< 0.001)**
Insufficient job control	46.8 ± 14.5	49.7 ± 11.4	1.486(0.139)
Interpersonal conflict	39.2 ± 10.5	39.8 ± 11.1	0.388(0.699)
Job insecurity	41.1 ± 12.6	46.7 ± 14.7	**2.676(0.008)**
Organizational system	51.5 ± 15.9	53.7 ± 15.0	0.930(0.354)
Lack of rewards	51.1 ± 14.8	55.2 ± 13.7	1.906(0.058)
Occupational climate	43.3 ± 15.2	42.8 ± 13.2	-0.231(0.818)
Total	45.4 ± 7.9	48.5 ± 7.3	**2.704(0.008)**

The final score of occupational stress ranges from 0 to 100, with higher scores reflecting higher stress levels. *p*-value was calculated using the Independent T-test.

### Effects of work-related musculoskeletal disorders on health-related quality of life

Table 4 shows the effects of work-related musculoskeletal disorders on health-related quality of life. There was a significant difference in the HRQoL of construction workers according to the presence or absence of WMSDs (*p* < 0.01). Furthermore, construction workers with WMSDs differed significantly in terms of Physical functioning (*p* < 0.001), Role physical health (*p* < 0.001), Vitality (*p* < 0.01), Social functioning (*p* < 0.05), Bodily Pain (*p* < 0.001), General health (*p* < 0.001), Physical component summary (*p* < 0.001), and Mental component summary (*p* < 0.05).

**Table 4:** Effects of work-related musculoskeletal disorders on health-related quality of life

Scales	Without WMSDs symptoms	With WMSDs symptoms	**t*(p)***
	Mean ± SD	Mean ± SD	
Physical function	97.8 ± 5.3	86.1 ± 13.3	**-7.875(< 0.001)**
Role physical	95.7 ± 12.3	77.1 ± 25.3	**-6.390(< 0.001)**
Emotional role	94.7 ± 14.3	93.8 ± 17.0	-0.407(0.684)
Vitality	51.5 ± 9.4	47.7 ± 10.0	**-2.646(0.009)**
Mental health	59.7 ± 8.9	61.8 ± 9.5	1.498(0.136)
Social function	71.6 ± 14.7	65.2 ± 15.2	**-6.601(< 0.001)**
Bodily pain	80.4 ± 16.3	63.8 ± 17.1	**-2.848(0.005)**
General health	66.8 ± 11.6	58.0 ± 12.1	**-4.891(< 0.001)**
Physical component summary	85.2 ± 7.8	71.3 ± 12.5	**-9.013(< 0.001)**
Mental component summary	69.4 ± 6.7	67.1 ± 7.5	**-2.139(0.034)**
Total	77.3 ± 6.3	69.2 ± 8.9	**-6.916(< 0.001)**

The final quality of life score ranges from 0 to 100, with higher scores reflecting higher quality of life. *p*-value was calculated using the Independent T-test.

The final quality of life score ranges from 0 to 100, with higher scores reflecting higher quality of life. *p*-value was calculated using the Independent T-test.

### Simple linear regression analysis of health-related quality of life

Table 5 presents a simple linear regression analysis of the health-related quality of life. A simple linear regression analysis was conducted to determine whether WMSDs affected HRQoL. Because F = 47.831 (*p* < 0.001), the regression model can be deemed suitable. R^2^ = 0.214, which indicates an explanatory power of 21.4%. The analysis demonstrated that WMSDs had a statistically significant impact on HRQoL. Specifically, the presence of WMSDs exerted a relatively greater effect on HRQoL compared to their absence.

**Table 5:** Simple linear regression analysis of health-related quality of life

	Β	S.E	*β*	t(*p*)	*F*(*p*)	R^2^
(Constant)	69.197	0.795		**87.053(< 0.001)**	**47.831(< 0.001)**	0.214
WMSDs	8.100	1.171	0.462	**6.916(< 0.001)**		

### Moderating effect of work-related musculoskeletal disorders in relation to occupational stress and health-related quality of life

**Fig. 1:**
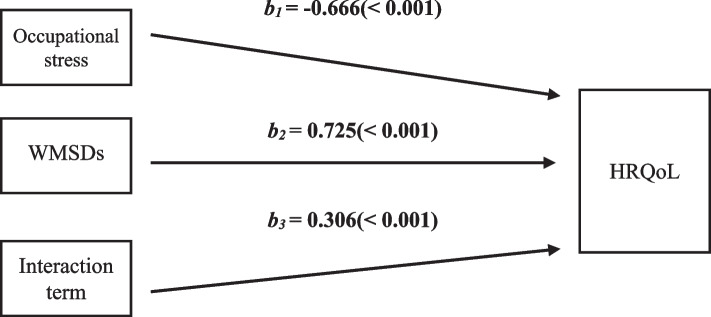
WMSDs moderating effect

The data were analyzed using the SPSS PROCESS macro (model) developed by Hayes (2013) to verify the moderating effect of WMSDs in the effect of occupational stress on HRQoL, and the results of the moderating effect analysis are shown in Fig. 1.

In order to analyze the moderating effect, the dependent variable was set as HRQoL, occupational stress (b1), WMSDs (b2), 'With' and 'Without' were coded as 1 and 2, respectively, and the interaction term (b3) of the two variables was simultaneously inserted and analyzed. The values, as outlined in Table 6, were obtained. From the analysis of the moderating effect, the main effects of occupational stress (*β* = -0.666, t = -8.517, *p* < 0.001) and WMSDs (*β* = 0.725, t = 6.547, *p* < 0.001) were evident when the first variables of occupational stress and WMSDs were input. Secondly, the interaction term between occupational stress and WMSDs was employed to verify if WMSDs had a moderating effect on the relationship between occupational stress and HRQoL, and a significant interaction effect was identified (*β* = 0.306, t = 2.765, *p* < 0.001). The explanatory power of the dependent variable by the input variables, indicated by R^2^, was 48.9%. Additionally, it was observed that the degree of change attributable to the interaction term had an explanatory power of 2.2%.

**Table 6:** Moderating effect of work-related musculoskeletal disorders in relation to occupational stress and health-related quality of life

Path						
Dependent variable (HRQoL)	Non-standardized coefficient		*β*	t(*p*)	LLCI	ULCI
	B	S.E				
Occupational stress(b_1_)	-0.754	0.089	-0.666	**-8.517(< 0.001)**	-0.929	-0.579
WMSDs(b_2_)	6.345	0.969	0.725	**6.547(< 0.001)**	4.432	8.258
Interaction term(b_3_)	0.347	0.125	0.306	**2.765(0.006)**	0.099	0.594
*R*(*R*^*2*^)	0.699(0.489)					
F	55.466**(< 0.001)**					
△R^2^ due to interaction	**0.022[F = 7.644(0.006)]**					

The omnibus test, which examines whether the change in R^2^ or the coefficient of the interaction term is meaningful, cannot explain the conditions under which the control variable interacts. Therefore, the interaction effect should be presented by replacing the specific value of the control variable (Aiken & West, 1991). Therefore, WMSDs, which are regulatory variables, were divided into "with" and "without" to check whether the simple regression line, which is the effect of occupational stress on HRQoL, was statistically significant. Consequently, as shown in Table 7, the simple slope of occupational stress on HRQoL was -0.666 (t = -8.517, *p* < 0.001) for with WMSDs and -0.360 (t = -4.593, *p* < 0.001) for without WMSDs indicating that both were statistically significant.

**Table 7:** The simple slope of occupational stress on HRQoL

WMSDs	The simple slope of occupational stress on HRQoL					
	Non-standardized coefficient		*β*	t(*p*)	LLCI	ULCI
	B	S.E				
With	-0.754	0.089	-0.666	**-8.517(< 0.001)**	-0.929	-0.579
Without	-0.407	0.089	-0.360	**-4.593(< 0.001)**	-0.583	-0.232

## Discussion

WMSDs refer to musculoskeletal injuries caused by work-related risk factors or work-related events [19, 20]; importantly, they are not the result of a sudden accident but rather a chronic outcome. Risk factors for WMSDs include inappropriate posture, long-term repetitive work, and handling of heavy objects. Moreover, excessive physical work is considered a major risk factor [21–24]; notably, psychosocial problems are also reported as risk factors for WMSDs [25].

### Prevalence of work-related musculoskeletal disorder and pain site

The results revealed that 53.9% of construction workers had experienced symptoms of WMSDs in the past 12 months. Moreover, an examination of WMSDs prevalence in Nigerian construction workers indicated a rate of 39.3%, while in Indian construction workers, the prevalence soared to 80.0% [8, 15]. Thus, it becomes evident that the prevalence of WMSDs within the construction industry exhibits some variation across countries. This difference may be due to the difference in the questionnaire form used in this study from the previous study. In the earlier study, pain areas were documented as 9, while in this study, they were recorded as 6. However, the questions asking about symptoms were similar.

In this study, construction workers most frequently reported lower back issues. Furthermore, an analysis of WMSDs prevalence by body part in the U.S. and Indian construction industries indicated that the back was the most commonly affected area [1, 8]. In Korean construction workers, physical factors such as inappropriate posture (tired or painful posture), heavy object handling (heavy object movement), and repetitive movements have been found to affect upper-extremity WMSDs [26]. The results of the present study may be due to the many repetitive tasks of lifting heavy building materials in inappropriate postures. Therefore, when designing musculoskeletal treatment and prevention programs in the workplace, areas with a high prevalence of WMSDs should first be considered.

### Effects of work-related musculoskeletal disorders on occupational stress

Based on the analysis of occupational stress associated with WMSDs in this study, the stress levels were 48.5 and 45.4 points for the groups with and without WMSDs, respectively, a statistically significant difference. WMSDs often also pose major threats to mental health and can be associated with an increased risk of developing other chronic health conditions [27]. According to a systematic review that investigated the connection between the psychosocial factors of construction workers and the presence of WMSDs, there was a reported correlation between WMSDs and at least one psychosocial factor. The most frequently reported factor was occupational stress [28]. In a study examining the factors that contribute to the increased prevalence of WMSDs in Italian workers, a higher level of work-related stress was significantly associated with an increased probability of experiencing WMSDs [29]. An analysis exploring the relationship between occupational stress and WMSDs among construction workers in India found a positive correlation. Specifically, it was posited that an increase in occupational stress could lead to the development of WMSDs [8]. The results of our study further substantiate the already-emphasized association between psychosocial factors and WMSDs in previous studies. Although it is not yet known precisely how occupational stress causes or increases WMSDs, it can strain muscles and cause musculoskeletal diseases; furthermore, it is believed to delay or worsen recovery from physical diseases by increasing awareness of symptoms and reducing coping skills [30, 31]. Therefore, establishing programs to manage occupational stress and WMSDs in the workplace effectively improves individual and organizational health by reducing the incidence and stress of WMSDs. The eclectic range of positive and healthy response mechanisms reported to mitigate stress in the construction industry was socializing, in addition to sports, hobbies, and more solitary pursuits [32]. Among them, doing activities outside of work such as hobbies is an effective tool to get a sense of pleasure after being bored at work [33].

### Effects of work-related musculoskeletal disorders on health-related quality of life

Analysis of total by HRQoL in this study showed that the scores were 69.2 and 77.3 points for groups with and without WMSDs, respectively, which was statistically significant. An analysis of the relationship between WMSDs' pain frequency and HRQoL (SF-36) among Norwegian industrial workers showed that the higher the pain frequency of WMSDs, the lower the HRQoL [34]. In addition, a study of Korean industrial workers on the impact of musculoskeletal symptoms on quality of life showed a negative correlation [35]. Our findings support the negative impact of WMSDs on HRQoL published in previous studies and suggest that WMSDs management is critical for enhancing HRQoL.

### Simple linear regression analysis of health-related quality of life

A simple linear regression analysis was conducted to determine whether WMSDs affected HRQoL. The analysis indicated that WMSDs exerted a statistically significant impact on HRQoL. Specifically, the effect on HRQoL was notably greater in the presence of WMSDs than in their absence. Moreover, a study analyzing factors affecting HRQoL among construction workers in India identified occupational stress and WMSDs as influencing factors [8], which is consistent with the results of the present study.

### Moderating effect of work-related musculoskeletal disorders in relation to occupational stress and health-related quality of life

This study corroborated the role of WMSDs in modulating occupational stress and HRQoL. Specifically, it reinforced the negative impact of WMSDs when occupational stress adversely affects the HRQoL of construction workers. Moreover, an analysis of the relationship between occupational stress and WMSDs among construction workers in India indicated that occupational stress was related to WMSDs [8]. Furthermore, an analysis of the effect of occupational stress on HRQoL in miners revealed that occupational stress affects HRQoL [36]. However, no prior study has investigated the moderating effect on construction and industrial workers. Therefore, a direct comparison with this study could not be made. Nevertheless, one of the most interesting findings of our study is the moderating effect of WMSDs. These results should be considered with caution and provide basic information for designing and managing WMSDs in the workplace.

### Limitations

The main advantage of this study is that it is possible to compare the effects of WMSDs on occupational stress and HRQoL of construction workers. Furthermore, the moderating effect of WMSDs was proved in the effect of occupational stress on the HRQoL of construction workers. Moreover, this was the first study to explore the moderating effect of WMSDs on the relationship between job stress and HRQoL. A limitation of this study is the failure to subdivide the construction jobs. Therefore, it is difficult to generalize these results to construction workers. Furthermore, as this was a cross-sectional study, it was insufficient to determine the impact of follow-up studies on the HRQoL of construction workers. Finally, excluding individual subjectivity and prejudice was difficult because they relied on self-reported reports through surveys. Future research should be conducted by subdividing the job field of the construction industry. In addition, it is necessary to study multidimensional aspects, including various variables such as exposure to physical working conditions, job requirements, and job satisfaction.

## Conclusion

WMSDs in construction workers have been identified as factors that significantly impact both occupational stress and HRQoL, suggesting that WMSDs exert a considerable control effect on occupational stress and HRQoL. Therefore, arbitration programs based on occupational stress and WMSDs are necessary to improve construction workers’ HRQoL. Moreover, to reduce occupational stress, it is necessary to help people enjoy their hobbies at the company's welfare level. Finally, to manage WMSDs, a system should be prepared to improve the physical environment of construction sites to reduce the physical burden.

## Data Availability

All data generated and analyzed during this study are included in the manuscript.

## Abbreviations

WMSDs: Work-related musculoskeletal disorder
HRQoL: Health-related quality of life
SF-36: Short-form health survey
